# Landscape change patterns at three stages of the construction and operation of the TGP

**DOI:** 10.1038/s41598-021-87732-8

**Published:** 2021-04-29

**Authors:** Ruikang Li, Yangbing Li, Bo Li, Dianji Fu

**Affiliations:** 1 Faculty of Geographical Science, Beijing Normal University, Beijing, 100875 China; 2 College of Geography and Tourism, Chongqing Normal University, Chongqing, 401331 China; 3 Key Laboratory of Surface Process and Environment Remote Sensing in the Three Gorges Reservoir Area, Chongqing Normal University, Chongqing, 401331 China; 4 The Research Center of Jinsha River Culture, School of Geographic Science and Tourism, Zhaotong University, Yunnan, 657000 China

**Keywords:** Ecology, Evolution, Ecology, Environmental sciences, Environmental social sciences

## Abstract

Analyses of landscape change patterns that are based on elevation and slope can not only provide reasonable interpretations of landscape patterns but can also help to reveal evolutionary laws. However, landscape change patterns and their model in different landforms of the typical watershed in the Three Gorges Reservoir Area (TGRA) has not been quantified and assessed effectively. As a complex geographical unit, the ecological environment in the middle reach of the Yangtze River has experienced great changes due to the construction of the Three Gorges Project (TGP) and its associated human activities. Here, based mainly on a digital elevation model (DEM) and remotely sensed images from 1986, 2000, 2010, and 2017 and by using GIS technology, speeds/ trends of landscape change, the index of landscape type change intensity, landscape pattern indices, and landscape ecological security index, the spatial and temporal evolution characteristics of different elevations, slopes, and buffer landscape types were analyzed in typical watersheds, as well as an evolutionary model of the landscape pattern. The results indicated that (1) the landscape types along with the land classification and buffer zone that were influenced by the TGR construction have undergone a phased change, with the period 2000–2010 being the most dramatic period of landscape evolution during the impoundment period; (2) landscape type shifts from human-dominated farmland to nature-driven forestland and shrub-land as elevations, slopes and buffer distances increased. The landscape has shifted from diversity to relative homogeneity; (3) land types and buffer zones played essential roles in the landscape pattern index, which is reflected in the differences in landscape type indices for spatial extension and temporal characteristics. The results of this paper illustrate the spatial–temporal characteristics of various landscape types at three distinct stages in the construction of the TGR. These findings indicate that the landscape ecological security of the watershed is improving year by year. The follow-up development of the TGRA needs to consider the landscape change patterns of different landforms.

## Introduction

More than 58,000 massive reservoirs had been built worldwide by 2015^1^ and approximately 45% of these had been constructed in China^2^. As an effective method for water resource utilization and regulation, dams have made important contributions to social and economic development. More rivers have been altered, and large-scale water conservation projects^3,4^ have been undertaken to satisfy the demands of rapid socioeconomic development^5,6^. As the greatest contributors to alter rivers’ natural properties, the construction of dams causing certain duress on watershed ecosystems to some extent, which can affect landscape distributions by impounding water for prolonged periods. The construction and operation of large reservoirs have profoundly changed the delivery of riverine material^7^, such as causing fragmentation of fish habitats, altering regional climatic environments, resulting in loss of species diversity, increasing soil erosion, and shifting the reservoir’s area water levels rhythm, and have caused unprecedented ecological and environmental challenges upstream and downstream^8^. In particular, the construction of master engineering has profoundly influenced landscapes. In recent decades, these changes have attracted extensive attention from experts and scholars around the world^9–12^. As especially frequent human activities, land inundation, flow manipulation, and fragmentation triggered by reservoir construction^13^ have crucial environmental impacts: unavoidable crop production losses^14^, changes in hydrological conditions^15^, soil erosion^16^, increases in population and pollution inputs^17^, which ultimately lead to changes in landscape patterns. In the context of global climate change, dam construction and its ecological effects are complex, potential, spatial, cumulative, and unpredictable^18^. Quantifying the landscape ecological impact of reservoirs is essential for developing appropriate strategies to reduce adverse impacts on regional environments. Land-use-based landscape ecological security assessments play an important role in the construction of ecological security patterns^19^.

For a start, it is necessary to distinguish the fundamental conceptions of land cover and land use. Land cover is generally defined as the coverage of the earth’s surface at present that has been formed by natural and anthropogenic influences^20^; land use is the consequence of long-term interactions between humanity and the natural environment. Land use reflects the utilization manner and condition of the natural properties of land by human beings. In other words, it means according to the natural characteristics of the land and under certain economic and social purposes, humans adopt biological and technological measures to manage and govern land in a long-term and cyclical manner^21^. Landscape refers to the mixture or repetition of regional ecosystem or land use type in a certain area of land^22^. From the perspective of Geography, it can be seen as geocomplex and land can be used as the landscape types in a sense, landscape-scale was considered as the suitable level to study the environmental impacts from human activities^23^. Landscape pattern refers to the spatial structure characteristics of the landscape such as spatial distribution, structure, and configuration of spatial components with a variety of sizes, shapes, and attributes^24^. It not only shows the heterogeneity of landscape but also reflects the result of natural or human disturbance. These patterns reflect the state of a region’s natural environment and influence the stability and order of the ecosystem^25^. With time, landscape elements are exceptionally resistant to spatial and temporal variability at multiple scales. The gradient effect is evident for different elevations, slopes, and topographic fluctuations^26^. Topographical factors, including elevation and slope, are among the many factors that are crucial to the natural environment^27^, which determines the changing direction and ways of landscape types to some extent. Geomorphic conditions are the most critical factor for determining the intensity of human transformations of landscapes. Landscape conditions form landscape evolution patterns that reflect not only the extent of human influence on natural ecosystems but also the responses of human activities triggered by landscape conditions^28^.

As one of the largest dam reservoirs in the world, the TGR is located in the middle reaches of the Yangtze River^29^ and is a fragile natural ecosystem^30,31^ with complex and rugged topography that play a big part in the evolution of landscape patterns and intentions of human disruptive activities^32^. The protection of the ecology and environment in the TGRA plays a crucial role in the green development of the Yangtze River Economic Zone^33^. With the construction of the Three Gorges Dam (TGD) and several increases in water levels, many areas upstream of the reservoir have been inundated^34^. Landscape patterns have also changed greatly with varying degrees of anthropogenic disturbance^35^. Anthropogenic activities like eco-migrants, urban construction, land use changes, comprehensive supporting facilities, and ecological engineering have resulted in changes in the structure and function of the ecosystem in the reservoir area^36^. Meanwhile, several measures taken to address agricultural surface pollution and mitigate soil erosion have also led to landscape element changes^37,38^. Besides, approximately three-quarters of the TGRA is mountainous, and approximately 24.74% of the area is steep and unstable with slopes greater than 25°^39^. In response to local ecological changes, the Chinese government has actively implemented various large-scale ecological policies and strategies, such as the Forest Projects^40^ and Special Water Management Plan^41^. These policies in China in the past directly affected the evolution paths of landscape types and landscape patterns in mountainous areas^42^.

In recent decades, there has been an abundance of national and international research on landscape evolution patterns, as is evidenced by qualitative and quantitative analyses using GIS and RS techniques in conjunction with landscape pattern indices^43^ that include the relationships between landscape and soil erosion^44^, ecological security patterns^45^, and granularity of landscape pattern effects^46^, among others. As research has progressed, some scholars have introduced landscapes into geography to study the relationship between landscape change and landforms^47^, and the scale of research has gradually shifted from comprehensive evaluations at large scales to in-depth studies at small scales. Moreover, most studies have lacked the dynamics of evaluation results over long periods. At this stage, many researchers have studied the evolution of landscape patterns in the TGRA before and after water storage; and have focused on spatial granularity effects^48^, effects of forest restoration on soil erosion^49^; and cropland patterns and driving forces^50^. Under the contexts of climate change, China’s “ecological civilization construction”, and economic and social transformation, how did the construction of the TGP affected the landscape element changes in the watershed? What are the differences in landscape evolution characteristics of distinct land types? These are research questions that we try to address.

While past work has mainly focused on landscape change and its evolution characteristics, little information is available on the relationship between landforms (based on elevations and slope gradients) and the evolution of landscape patterns. Landscapes can vary under different landform conditions^47^, and the landform context had a drastic impact on landscape pattern evolution and human disturbance^32^. The land cover and landscape types in the TGRA have shown great variations due to the influence of water conservancy construction. Therefore, this paper aims to investigate the evolution of each landscape pattern based on elevation and slope zone reclassification in typical watersheds of Chongqing under the context of the TGP construction and operation. The intention is to provide referential significance for land use planning, the establishment of ecological patterns, and environmental protection in the core of the TGRA.

## Background: construction timeline of the TGP

**Figure 1:**
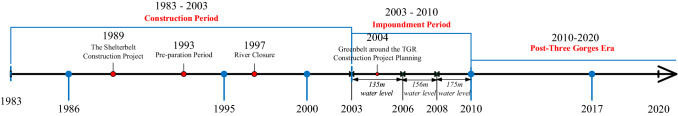
Construction timeline of the TGP.

In China, the TGP began in 1993 and was completed in 1997 on the upper trunk of the Yangtze River^50^, which is the largest hydroelectric project ever conducted^51^, to accommodate flood control, irrigation, increased navigation, and power generation needs^52,53^. Hydropower construction was completed in 2002^39^, reservoir filling was initiated in 2003, and impoundment was completed in 2010^54^. By June 2003, the water level was expected to increase to 135 m ASL (above sea level), and the first filling stage was completed in 2003^55^. The reservoir level reached 156 m ASL in October 2006. In October 2010, the TGR reached its normal storage level of 175 m ASL for the first time^56^, and approximately 240 km^2^ of citrus and farmland was inundated. Water levels of the TGR are maintained between 145 m ASL from October to March and at 175 m ASL from April to September^57,58^. By the end of 2010, the TGP had been completed, and the entire reservoir area entered the Post-Three Gorges era^32^. The formation of the TGRA is a direct consequence of the dam^59^. According to previous relevant studies, the TGRA can be divided into three critical moments: the construction period, impoundment period, and Post-Three Gorges era^60,61^. Considering the actual situation of the hinterland basin of the reservoir area and the difficulty of data acquisition, this study is divided into three phases: 1986–2000, 2000–2010, and 2010–2017 (Fig. 1).

## Materials and methods

**Figure 2:**
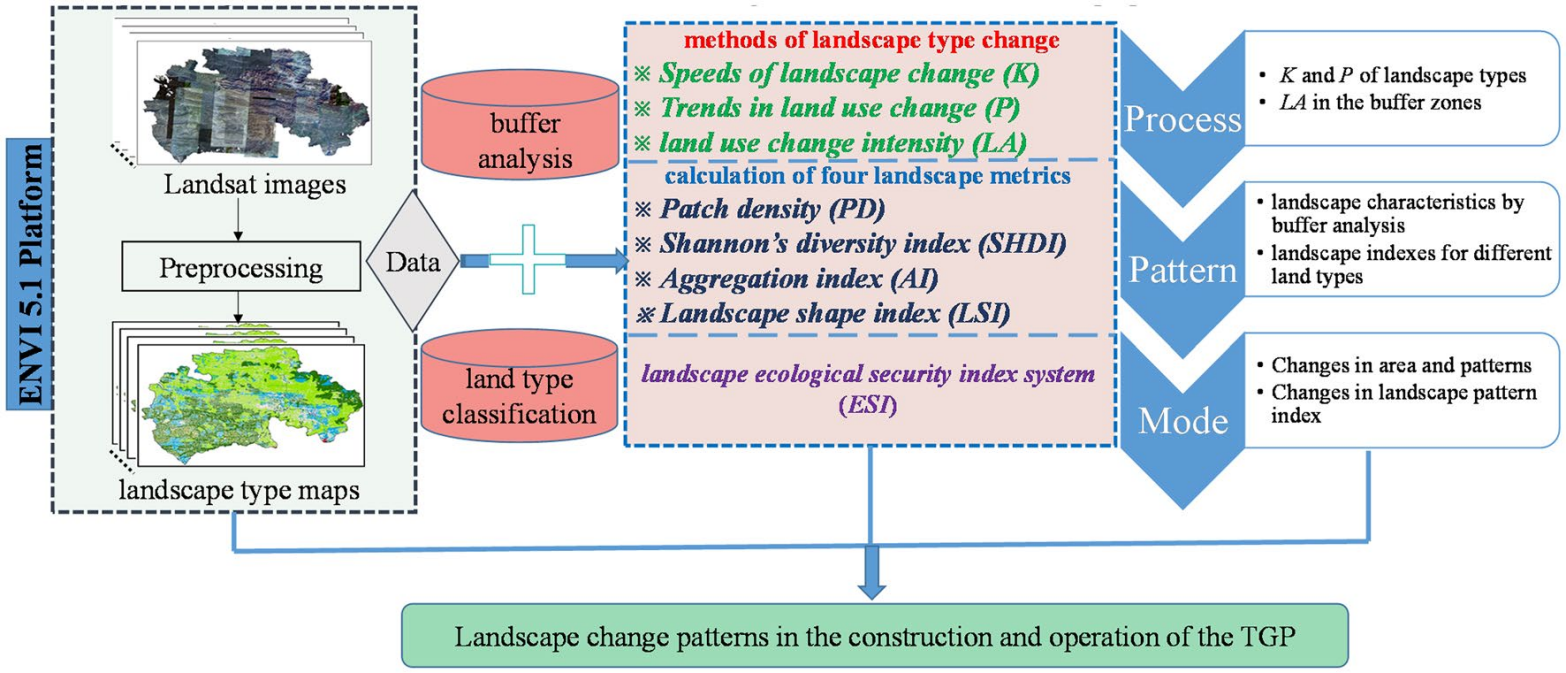
Flowchart for the evolution of landscape change patterns in this study.

The workflow chart for the evolution of landscape change patterns in this study can be summarised as follows (Fig. 2). First, the landscape type maps were classified by ENVI 5.1 platform. Second, based on buffer zone analysis and land type division in ArcGIS 10.2, the methodological evaluation system of this paper is constructed from the methods of landscape element change, calculation of landscape pattern index, and landscape ecological security index. In the results analysis part, we first analyze the *K* (Speeds), *P* (Trends) of landscape types and *LA* (Index of landscape type change intensity) in buffer zones; then the landscape metrics (*PD*, *SHDI*, *AI*, and *LSI*) were computed to reveal the landscape characteristics in buffer zones and land types; finally, combined with the method of landscape ecological security index, we summarize the change modes in area and landscape pattern.

### Study area

**Figure 3:**
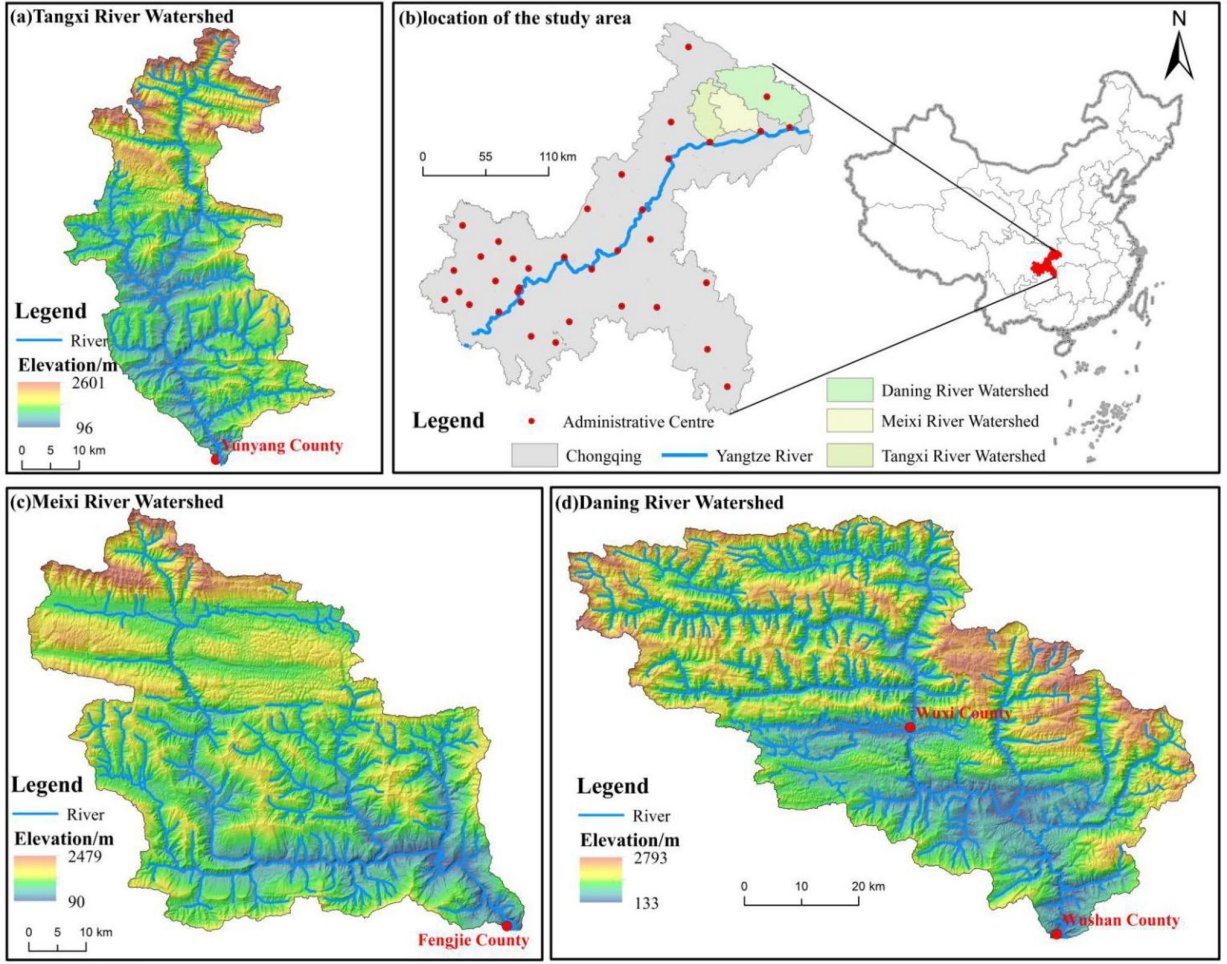
Location and topography of the study area in the TGRA. Maps were generated using ArcGIS 10.2 for Desktop (http://www.esri.com/sofware/arcgis).

Tangxi River watershed (TR), Meixi River watershed (MR), and Daning River watershed (DR) in the hinterland of the TGRA were used as case studies (Fig. 3). The study area extends over 31°02′39″ ~ 31°44′01″ N, 108°37′ ~ 110°09′05″ E with a total area of 7938 km^2^. It has a northern subtropical humid monsoonal climate, and the prevailing soil types are yellow–brown (Similiar to Luvisols in FAO/Unesco) and purple soils (Similiar to Regosols in FAO/Unesco)^62,63^. This area belongs to the Chongqing section of the TGRA, covers four counties (Yunyang, Fengjie, Wushan, and Wuxi) and is the core area for ecological protection and development in northeast Chongqing and is also a fragile and ecologically sensitive area of the TGRA with high mountains and steep slopes^17^. The watershed belongs to the first tributary of the north bank of the Yangtze River; TR, MR, and DR are adjacent to each other with various natural social and economic backgrounds. DR is a karst watershed, MR is combined karst and non-karst watershed, and TR is heavily influenced by coal and other industrial wastes with many factories, coal mines, and construction sites. Our study area is representative of the contemporary landscape and is subject to natural and anthropogenic gradients and disturbances.

### Data source and processing

**Figure 4:**
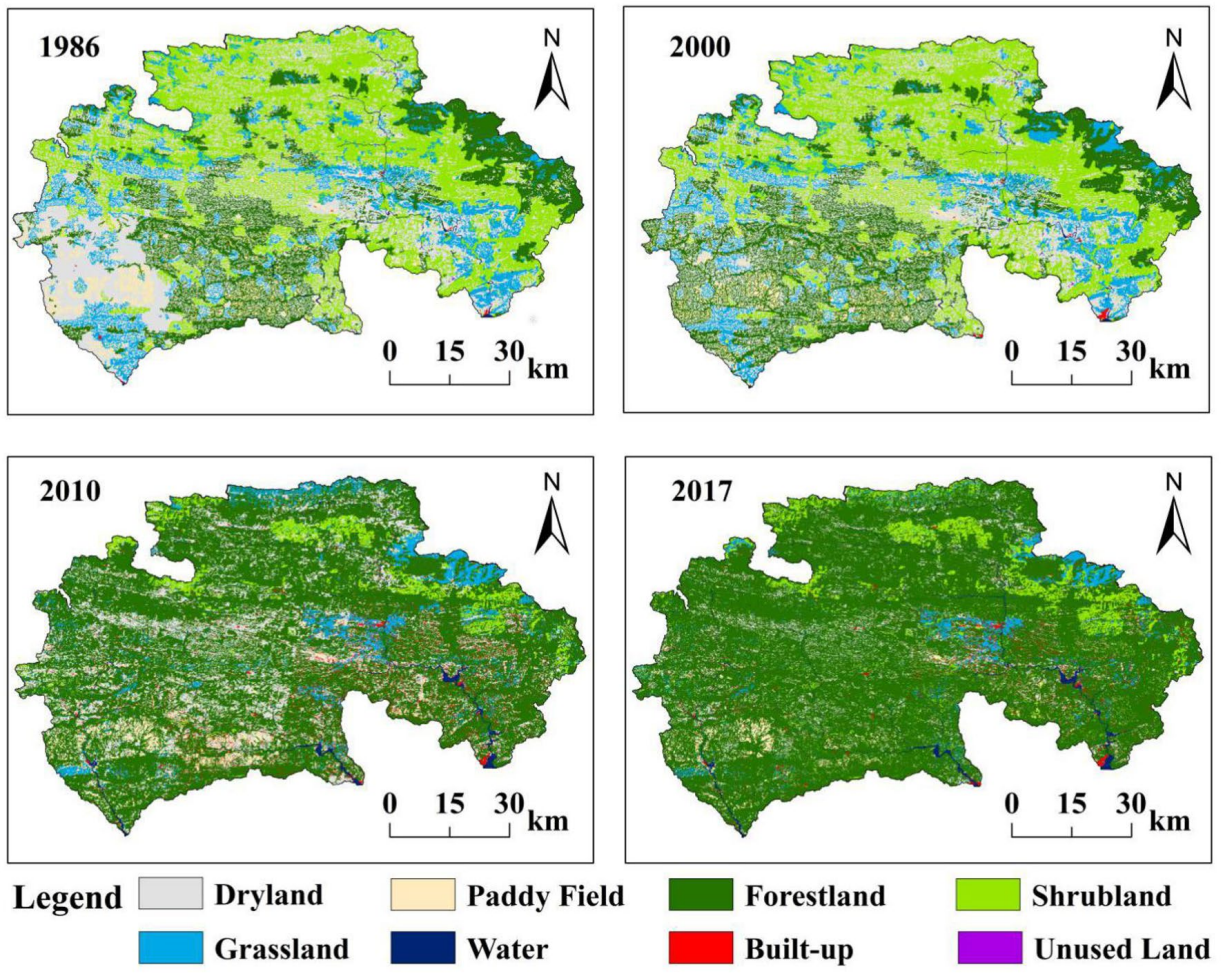
The spatial division of landscape type in the watershed from 1986 to 2017. Maps were generated using ArcGIS 10.2 for Desktop (http://www.esri.com/sofware/arcgis).

Our basic data sources include multitemporal satellite datasets and a DEM. (1) Landscape type maps for 1986, 2000, 2010, and 2017 (Fig. 4) were obtained from Landsat Thematic Mapper (TM) and Enhanced Thematic Mapper-plus (ETM +) images with 30 m resolution using nonlinear classification and artificial visual interpretation methods. In this paper, the support vector machine method is used in landscape classification and alters manually the misclassified landscapes. By verifying the data accuracy through the confusion matrix and ensuring the Kappa index is above 0.80. The regional landscapes were divided into dry land, paddy field, forestland, shrubland, grassland, water area, built-up areas, and unused land^64^. To meet the needs of the study, land use data from 1986, 2000, and 2010 were rigorously compared with data provided by the “data center for environmental and ecological sciences in western China” of the National Natural Science Foundation of China. For the 2017 land-use data, we strictly compared them with the high-resolution images from resource satellites with a resolution of 2.5 m during the interpretation process. Currently, we are using a combination of random sampling checks and field surveys to ensure data accuracy, and both have an accuracy rate of 92%, which meets the needs of this study. (2) Elevation and slope maps are based on 30 m resolution DEM data, which were downloaded from the CAS Resources and Environmental Science Data Center (http://www.resdc.cn). (3) Watershed vector boundaries were extracted from the DEM data with the Hydrology toolset in ArcGIS 10.2.

## Results analysis

### Changing speeds (*K*) and trends (*P*) of landscape types for each land type

**Figure 5:**
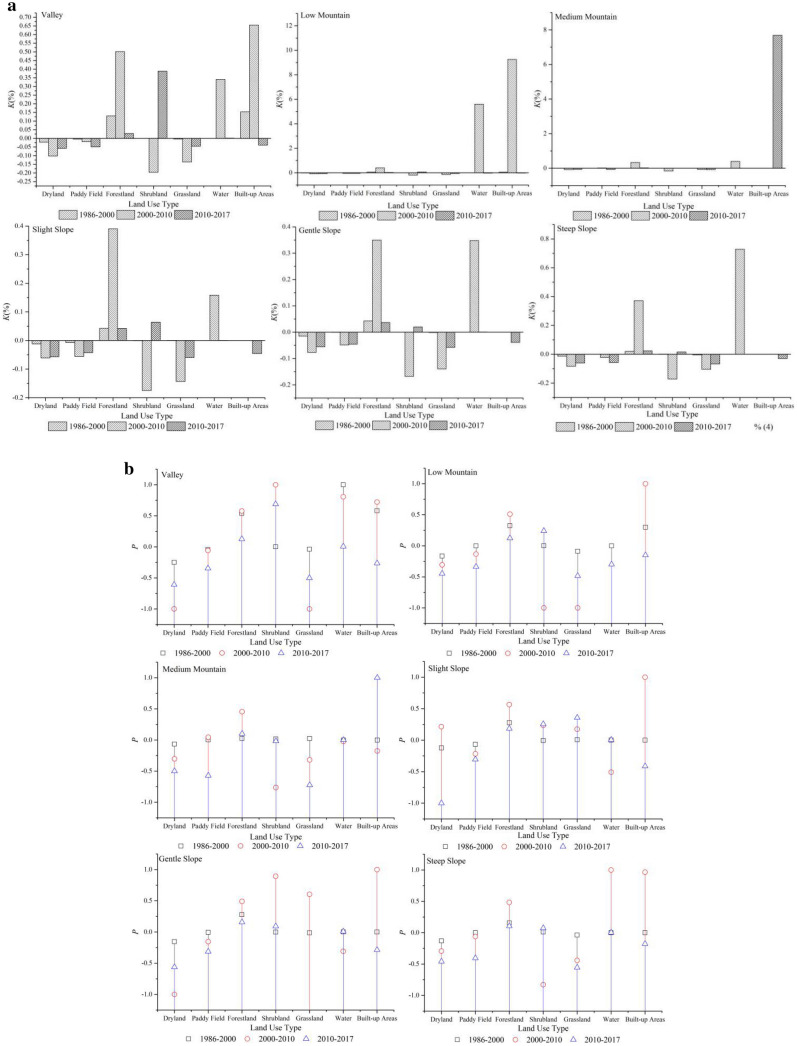
(**a**) Speed of different land cover for different landforms of the watersheds, (**b**) The trend of different landscape types for different landforms of the watershed.

We overlaid the landscape type map with the landform classification data by using the Intersection tool in ArcGIS 10.2 software to obtain the landscape types for a variety of land types and then calculated the *K* and *P* values based on the methods described in Formula (1) and (2), and the results are shown in Fig. 5.

Figure 5a exhibits the changes in *K*-values along with landforms for each landscape type. From 1986 to 2000, the *K*-values of each landform did not change apparently while the area of forestland increased prominently in valleys with slopes within 15°. From 2000 to 2010, the *K*-values for altitude and slope changed differently. Waters and built-up areas showed clear increasing trends at elevation gradients below 1000 m, while forestland and waters showed the most pronounced increases in each slope zone. This dramatic increase was mainly due to the rise in reservoir levels and the implementation of the national migration policy. As a result of urbanization, the total amount of built-up areas exhibited an upward trend from 2010 to 2017 in the middle mountainous areas where the elevations were higher than 1500 m.

Figure 5b represents the trend of *P* in landscape type along with land classification at each time point. In the period 1986–2000, the landscaping trend changed slightly except in valley areas. In this region, forestland, water, and built-up areas all expanded in an “upward” fashion. From 2000 to 2010, land use trends varied for elevation and slope. Cultivated land increased for slopes less than 8°; in areas below 1000 m elevation and with slopes less than 15°, settlements were the dominant landscape, and in both low and steep slope areas, water sources increased greatly as the water level rose. From 2010 to 2017, the landscape trends changed slightly, except in the valley and mid-mountain areas. In the valley areas, shrublands expanded in an “ascending” fashion. For the mid-hills, the built-up area expanded remarkably in an “ascending” state.

In general, landscape types in the watershed change at different rates and trends along elevation, slopes, and buffers, and the changes are phased and exhibit a process that changes from quantitative to qualitative. The period from 2000–2010 experienced the most intense landscape evolution during the impoundment period.

### Analysis of changes in the level of land use degree (*LA*) in the buffer zones

**Figure 6:**
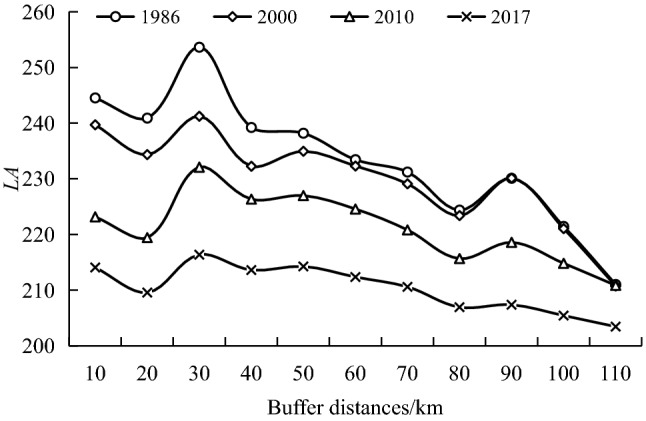
Comprehensive landscape degree index in the buffer zone of the watershed.

**Figure 7:**
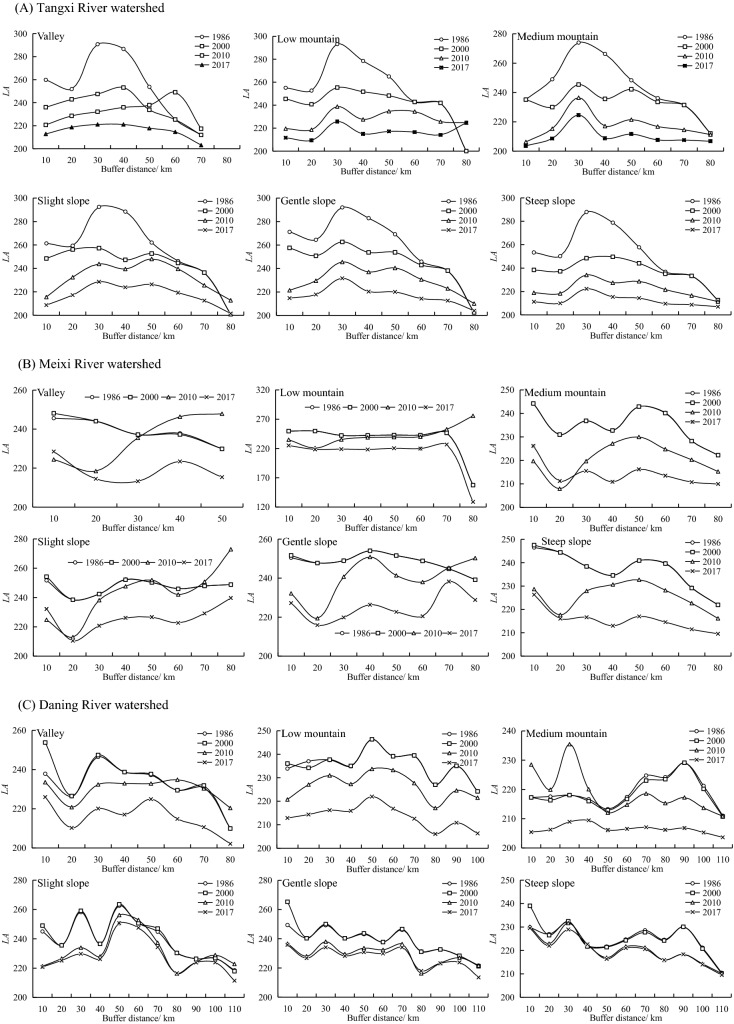
Comparison of *LA* in different buffer zones of watersheds from 1986 to 2017.

We used the Intersect tool in ArcGIS 10.2 software to overlay the landscape types of different landform maps with the watershed buffers and then calculated the index of landscape type change intensity (*LA*) values based on the method described in Formula (3), and the results are shown in Figs. 6 and 7.

Variations in buffer distance reflect some extent the variations in upstream and downstream distances. Figure 6 shows that the *LA* of each buffer zone in the basin showed a general downward trend that reached a maximum value for a buffer zone of 30 km and a minimum value for a buffer zone of 110 km. Within the 30 km buffer zone, the terrain is relatively flat, dominated by micro-slopes, and influenced by the intensity of human activities, while the degree of development is relatively high.

As illustrated in Fig. 7, the landscape changes in different land types in buffer zones of the watersheds varied from 1986 to 2017. Within the 30 km buffer, all land types in the TR had the highest landscape synthesis with reduced fluctuations around them, while the landscape synthesis in the MR and DR buffers showed notably different features across the different media. For the MR, the largest number occurred for a buffer zone of 10 km during the period of 1986–2000 among each geomorphic type. The 50 km buffer zone was largest in 2010 for the valley, medium-mountain, and steep slope areas, while the 80 km buffer zone was largest for the low-mountain, slight slope, and gentle slope areas. Unlike 2010, the *LA* of the MR showed an “N” type trend in elevation with the increase of buffer distance, and a “W” type trend in slope. This illustrates that the spatial distribution of the *LA* in the MR showed an obvious spatial heterogeneity in terms of elevation and slope. In 2017, except for the gentle slope, the 70 km buffer zone was the largest, while the other geomorphic areas were all largest in the 10 km buffer zone. The maximum value of the *LA* among the geomorphic areas in the DR occurred in different buffer zones. By comparing relevant research results, it was found that the 30 km buffer zone in the TR, 50 km buffer zone in MR, and 10 km buffer zone in the DR were mainly 800–1000 m ASL, and the lithology was mainly sandstone and mud shale, which are easily reclaimed for farmland. Human activities were relatively concentrated^65^, so *LA* was relatively high.

### Four landscape indexes (*PD*, *AI*, *LSI*, *SHDI*) characteristics by buffer analysis

**Figure 8:**
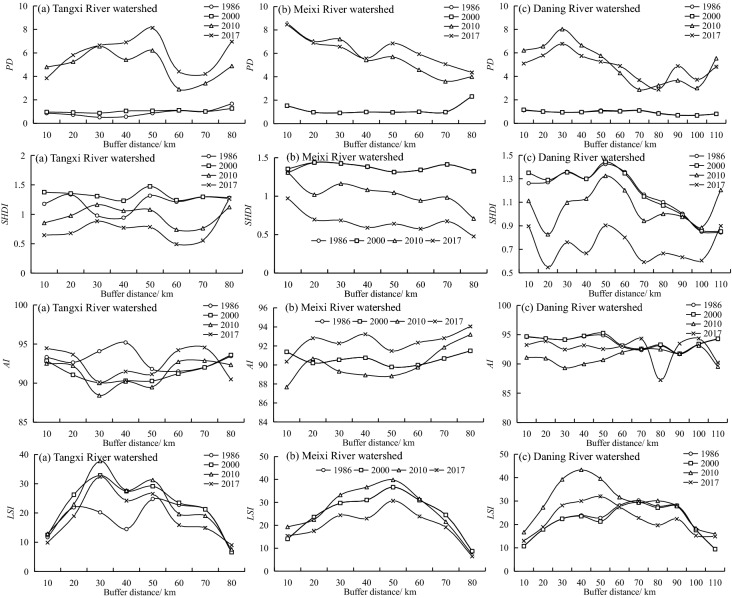
Changes in landscape metrics along with the buffer distance of the watershed from 1986 to 2017.

We overlaid the landscape type map with the buffer data using the Intersect tool in ArcGIS 10.2 software to obtain landscape types with different buffers and then calculated *PD, SHDI*, *AI,* and *LSI* values on the FRAGSTATS 4.2.1 platform; and the results are shown in Fig. 8.

Figure 8 shows that *PD* and *AI* both increased at four-time points, while *SHDI* and *LSI* decreased. In particular, from 1986 to 2000, the *PD* values in all three watersheds were less than 2, and these small values were less affected by the construction of the TGP. After 2000, due to the implementation of ecological migration and circular economic development, there was an obvious increase in *PD* values and decrease markedly in *SHDI* and *LSI* values. With increasing buffer distances, *PD* showed an appreciable upward trend in MR and DR and increased volatility in TR. During the study period, *SHDI* showed a decreasing trend as the buffer distance increased. This indicates the transition from a diverse landscape of cultivated land, forest, shrub, and grass to a single landscape dominated by forestland in the watershed with the advent of the Post-Three-Gorges Era. For *AI* values over 90, landscape aggregation is evident. With increasing buffer distance, the *LSI* showed an inverted U trend. In 2017, the *AI* and *PD* in the DR show the lowest value in 80 km buffer zones, this indicates the highest degree of landscape fragmentation in this zone.

Overall, the landscape pattern characteristics in the watershed showed a noticeable change as a result of TGP progression, with an upward trend in *PD* and the downward trend in *SHDI*, *AI,* and *LSI*. This may imply an increase in ecosystem quiescence.

### Characteristics of landscape indexes (*PD*, *AI*, *LSI*, *SHDI*) for different land types

**Figure 9:**
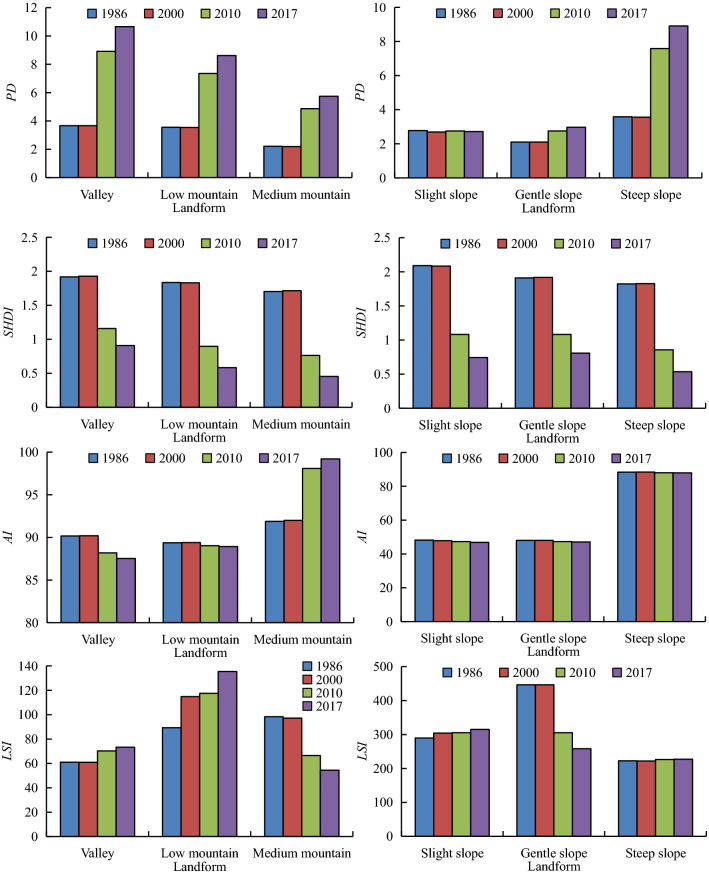
Changes in landscape index along with landforms from 1986 to 2017 in the watershed.

The different trends of the different landscape indices are shown to be under the influence of major water projects and rapid urbanization (Fig. 9). From 1986 to 2017, there was a distinct increase in the *PD* index, a wave-like downward change in the *SHDI* and *LSI* indices, and a less pronounced change in the *AI*. These changes indicate that the fragmentation degree increased, the landscape multiplicity decreased, and the landscape shape was relatively normal with the progression of the TGP. The landscape indices of different landform were obvious distinctions.

Specifically, *PD* tended to increase with elevation and decrease with slope along with greater fragmentation of valleys and steep slopes; *SHDI* decreased with elevation and slope, and the ecosystem may be more homogeneous; *AI* decreased first and then increased with elevation and slope, respectively, with greater aggregation of elevation than slope; *LSI* increased first and then decreased with elevation and slope while showing “N”-shaped changes. The landscape patterns were more complex and irregular in low mountains and gentle slopes.

### Characteristics of changes in landscape types and landscape indicators before and after the impounding period

#### Changes in area and patterns of landscape types over three time periods

**Figure 10:**
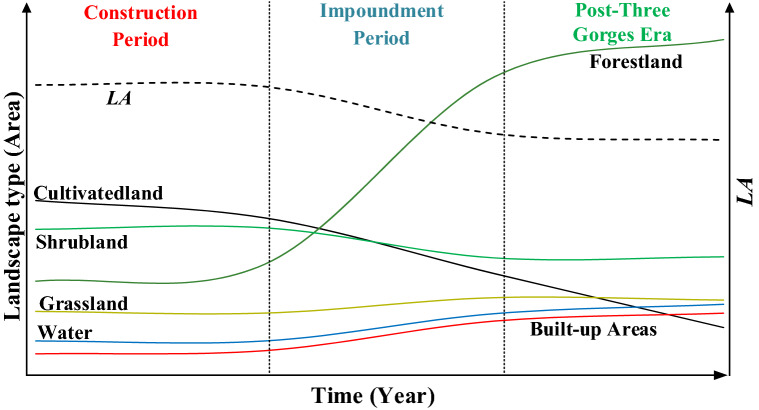
Change of landscape types in the watershed.

From the mid-1990s to the present, the landscape of the TGRA has undergone dramatic changes due to the construction of the Three Gorges Hydropower Station (TGHS), which has attracted much attention^38^. Due to intense and complex human pressures, the landscape structures and ecosystem service functions in the region have experienced frequent changes over the last 30 years. A large amount of land has been flooded due to the construction of hydroelectric dams and infrastructure. The relocation of immigrants and redevelopment of towns and cities have led to a dramatic decrease in the area of water in cultivated land and experienced a deceleration in growth, which is very consistent with the results indicated by the previous studies^30^. The conversion of cropland to forest has been the dominant landscape transformation process^49^, the area of cropland around the TGRA is decreasing^30^, and approximately 28% of farmland in China’s mountainous counties is abandoned^66^. Landscape change is phased^67^, and long-term stable landscape transitions can be used to reveal models of landscape evolution (Fig. 10).

In the first period (before 2000), there were conspicuous inter-conversions between different landscape types^39^ with concentrated and contiguous agricultural land forming the main landscape type of the watershed. The composite index of landscape extent was higher than in the other two periods, and the trend of landscape ecological security was improving.

The direct impact on the watershed during the impoundment period from 2000 to 2010 refers to the conversion of cultivated land to water bodies^68^. During this period, landscape changes manifested as an increase in forestland due to the implementation of natural reserves and government forest projects. Arable lands with slopes greater than 25° were best converted to the forest in accordance with the arable land conversion policy^40^. At the same time, due to land abandonment, arable land was converted to grassland or other landscape types. The general trend of landscape change was from low-cover types to high-cover types. The overall ecological security situation has declined due to the equal emphases on development and conservation.

At the present stage (after 2010), forests form the main landscape type of the watershed. As a result of China’s policies of “ecological civilization construction” and targeted poverty alleviation, farmers earn income by transforming their agricultural production methods to achieve unity of economic and ecological benefits^69^. The ecological security situation of the landscape shows a favorable development trend.

**Figure 11:**
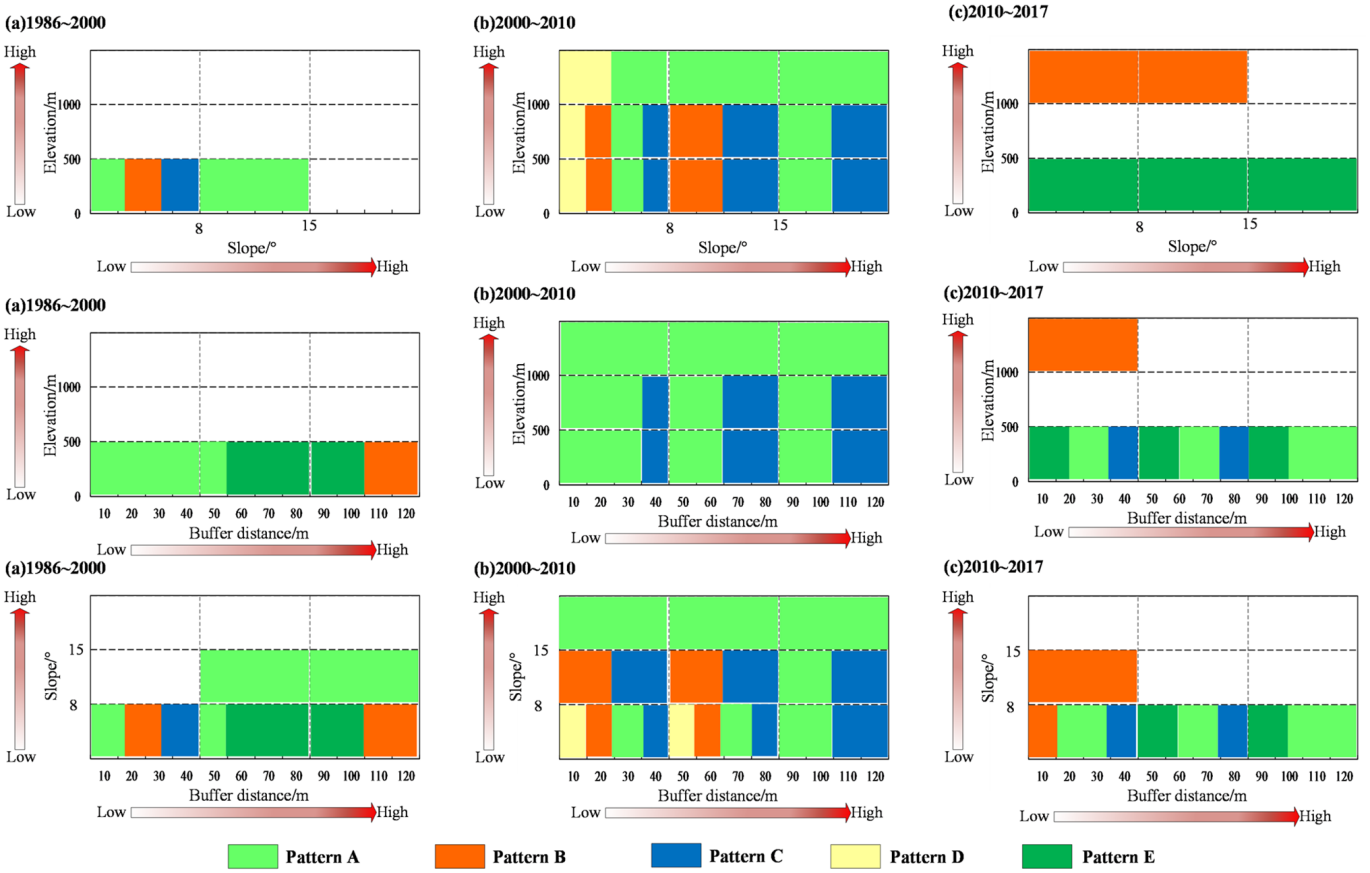
Landscape evolution model in a typical watershed of the TGRA. Pattern A: conversion of other landscape types to forestland; Pattern B: conversion of other landscape types to built-up areas; Pattern C: conversion of other landscape types to water bodies; Pattern D: conversion of other landscape types to farmland; Pattern E: conversion of other landscape types to shrubland.

The landscape is affected by complex topographic variables such as elevation and slope. Kelarestaghi^70^ found that altitude and slope are the physically effective factors that drive landscape change. Given previous work, we hypothesized that the watershed contained five patterns of landscape evolution with increasing elevation, slope, and buffer zone width under the influence of the TGP from 1986 to 2017 (Fig. 11). During the first period from 1986 to 2000, patterns A, B, C, and E occurred in valleys with slopes less than 15°, all buffer zones with altitudes less than 500 m, and slopes less than 8°. From 2000 to 2010, the landscape patterns of all spatial regions, especially those regions with slopes less than 15° and altitudes less than 1,000 m, were deeply influenced by human activities, and the main patterns were Mode A and Mode C. Unlike the previous phase, Mode A was distributed in the slight slope belts within the 50 km buffer zone due to relocation and urban renewal. From 2010 to 2017, the landscape of the whole spatial region for Modes A, C, and E was especially located in areas with slopes less than 8° and elevations less than 500 m. Model B was distributed within a 40 km buffer zone with slopes less than 15° and elevations greater than 1,000 m.

#### Changes in landscape pattern index over three time periods

**Figure 12:**
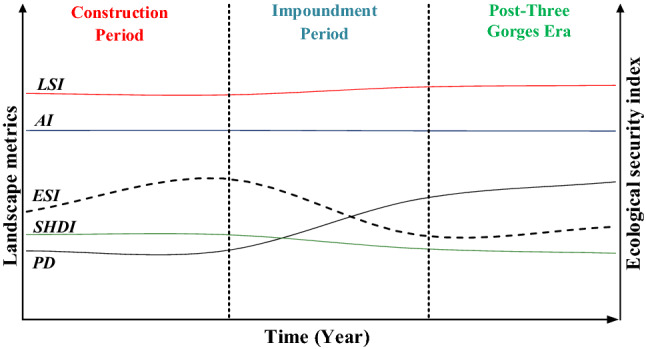
Change in landscape pattern indexes in the watershed.

Landscape types exhibited a distinct transformation phase before and after impoundment and are sensitive to elevation and slope^39^. In this context, the changes in the landscape pattern index in this study were divided into three periods during the construction of the TGR, just as shown in Fig. 12. In the first stage (before 2000), the landscape pattern index change was not obvious, the transition of landscape categories was stable, and the landscape pattern was reflected by the reclamation of agricultural land, which increased rapidly^35^. In the impoundment period from 2000 to 2010, the *PD* index exhibited a remarkable increasing trend, *SHDI* showed a decreasing trend, and the changes in the AI and LSI indices were not obvious. For the cases of economic development and ecological protection, a landscape pattern with high heterogeneity and low diversity was present, with an increase in forest and water area being the most notable landscape dynamic pattern. In recent decades, (after 2010), the *PD* index has dramatically increased, the *SHDI*, *AI* and *LSI* indices have not changed notably, and the landscape has shifted from diversified to relatively unitary.

From 1986 to 2017, the *ESI* showed a trend of N, which indicated that the spatial structure of the landscape was more stable and ecological security was more reasonable due to the implementation of policies such as the return of farmland to forest and designation of red lines for ecological protection. Due to the disturbing effects of the water conservancy construction itself, it was more difficult to restore the ecosystem to its preconstruction state. As the most basic and important type of landscape, both the natural environment and human activities can tremendously trigger landscape differences. The study of landscapes and their mechanistic driving factors are conducive to the optimization and improvement of policy mechanisms.

The changes in landscape types and landscape pattern indices at different stages are consistent with the laws and needs of reservoir construction. Over time, the spatial structure of the landscape becomes more stable and ecologically safe.

## Discussion and Conclusion

### Discussion

Over the 1986–2017 period, the arable landscape area of watershed decreased in each landform, which is in agreement with the findings of MEE (2018)^71^. We have shown that the landscape in the watershed changed from arable land to forestland, and Xu et al. (2020)^72^ found that cropland area in the TGRA has dropped from 29.0% in 1997 to 22.9% in 2016. Our finding supports the contention that ecological functions have been improved (SAGCAE, 2010)^73^, however, landscape ecological security can hardly revert to that before dam construction. Our study considered topographical factors are the foundation of landscape pattern formation and their spatial characteristics influence the evolution process of the landscape. Based on the elevation and slope characteristics, we divided the whole watershed into six categories and presented the landscape change pattern at each land type. In addition, we analyzed the differences of *LA* and landscape indexes of each land type for three different periods with the increase of buffer distance.

There are some limitations in our study that deserve mention. Firstly, it is difficult to precisely quantify modes of landscape change patterns in watersheds. Secondly, due to the complexity of the data acquisition on socioeconomic aspects of the watershed, this paper failed to reveal the driving mechanisms of the evolution of landscape patterns in the watershed. Lastly, we did not consider the watershed in the head and tail of the TGRA due to the lack of monitoring data.

### Conclusion

In the context of China’s “ecological civilization construction”, considering the noticeable impact of multiple stressors of TGP construction on ecological changes, this paper analyzes the changes in the degree of landscape evolution through measures related to the analysis of landscape type changes and landscape patterns in typical watersheds of the TGRA from 1986–2017 and draws the following conclusions.

Changes in the landscape patterns of the watershed are closely related to the construction of the TGP. During the construction period from 1986–2000, landscape types and landscape patterns change insignificantly, with arable land being the dominant landscape transformation process. From 2000 to 2010 (i.e., impoundment period), land use showed dramatic changes, with the conversion of arable land to forestland being the dominant landscape transformation process, water area changes were not obvious, and landscape types showed different trends in different regions. The results indicate that ecological conservation policies have a greater impact on landscape type change than reservoir impoundment. After 2010, landscape types changed more in areas with elevations below 500 m and slopes below 8°. Changes in land use type in the watershed brought landscape changes, and landscape fragmentation and diversity showed increasing trends throughout the study period.

With the increase of buffer distance, *LA*, *PD*, *SHDI*, *AI*, and *LSI* show negligible differences within different land types. *LA* of each buffer zone in the basin showed a general downward trend that reached a maximum value for a buffer zone of 30 km and a minimum value for a buffer zone of 110 km. Due to the complexity and diversity of the geographic environment in three watersheds, the distribution of *LA* has obvious spatial heterogeneity. As for the four landscape indexes, the landscape pattern characteristics in the watershed evidently changed as a result of TGP progression, with an upward trend in *PD* and the downward trend in *SHDI*, *AI,* and *LSI*. From 1986 to 2017, the *ESI* showed a trend of *N*, which indicated that the spatial structure of the landscape was more stable and ecological security was more reasonable due to the implementation of policies such as the return of farmland to forest and designation of red lines for ecological protection.

In this study, we employed GIS and RS tools, buffer analysis, and calculations of *K*, *P*, *LA*, and landscape pattern indices to analyze the spatial–temporal differential characteristics of the regional landscapes that were induced by dam construction and reservoir impoundment. In view of the phase of the large water conservancy project and the particular properties of the watershed and combined with the classification of land types based on elevation and slope gradient, our study reveals the landscape ecological effects over the long term sequenced from the perspectives of speed, tendency, intensity, and landscape pattern. Reservoir construction is an important human activity, and relevant studies of landscape models and patterns before/after reservoir construction can help us understand the generalizations of regional sustainable development. The landscape pattern in the riparian zone of the Three Gorges Reservoir and its response to ecological safety will be studied in the future.

## Methods

### Elevation, slope reclassification, and land types classification

Watershed elevations were reclassified into < 500 m, 500–1000 m, and > 1000 m based on geomorphological classification criteria using 30 m spatial resolution DEM data. Slope (degrees) was extracted from the DEM. Using the Slope Spatial Analysis tool in the ArcGIS software, we obtained a rasterized slope map of the study area. The slopes were reclassified into three grades of < 8°, 8° ~ 15° and > 15°^74,75^. Based on the elevation and slope characteristics, the land types were divided into six main categories, with watershed classifications and their area percentages shown in Table 1. Watersheds are mountainous and are present where more than 87% of the area has a topographic slope of more than 15°.

**Table 1:** Elevations, slope reclassifications, and land type classifications and their area percentages (%).

Elevation zone/m	Geomorphologic classification	Area percentage	Slope zone/°	Slope classification	Area percentage
< 500	Valley	11.32	< 8	Slight slope	3.72
500–1000	Low Mountain	37.15	8–15	Gentle slope	8.76
> 1000	Medium Mountain	51.53	> 15	Steep slope	87.52

### Landscape spatial analysis: buffer analysis

**Figure 13:**
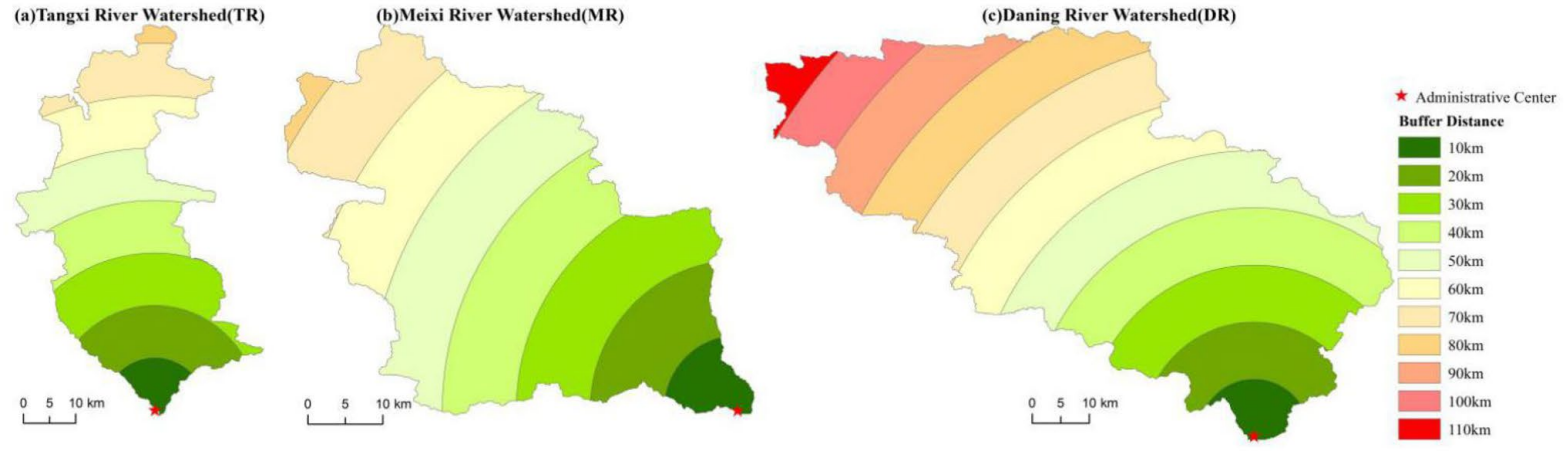
Setting up of watershed buffer zone. Maps were generated using ArcGIS 10.2 for Desktop (http://www.esri.com/sofware/arcgis).

As the basic contents of GIS spatial analysis, buffer analysis is the effective vehicle to describe the impact of geographical objects on their surroundings and to solve the problem of spatial proximity^76^. It is to create a faceted area around the analysis object at a certain distance to identify the radiation or influence of the analysis object on neighboring objects. To reflect the changes in landscape types and landscape metrics at different distances from the mainstream of the Yangtze River, this study analyzed a buffer zone with a radius of 10 km, which was centered on the outlets of the lower reaches (county government locations) of the three watersheds, to create a buffer zone, as shown in Fig. 13. The generated buffer zone was used to segment the landscape type status map to obtain landscape type maps of different buffer zones for each period. The land-use change velocity index (*K*), land-use change trend (*P*), and land use degree index (*LA*) were calculated by overlay analysis to analyze land use changes in different elevations and slope zones. This paper then explores the patterns of land use evolution in different buffer zones over different periods in the watershed.

### Data analysis methods of landscape type change

#### Speeds of landscape change (*K*)

The single land use dynamic degree (*K*) is used to reflect the rate of change for a certain landscape type and the differences between each type^38,55,77^, the mathematical expression is as follows:

1 $$K = \frac{{U_{t2} - U_{t1} }}{{U_{t1} }} \times \frac{1}{t2 - t1} \times 100{\text{\% }}$$

where $U_{t1}$ is the area of a land use type at time $t1$ and $U_{t2}$ is the area of the land use type at time $t2$; if $t$ is set as one year, the value $K$ is the annual comprehensive change rate of land use in the watershed.

#### Trends in land use change (*P*)

In our study, a single land use spatial change trend model was used to reflect the changing trend of landscape types^78^. The basic equation is shown below:

2 $$P = \frac{{U_{t2} - U_{t1} }}{{\Delta U_{out} + \Delta U_{in} }}$$

where $P$ denotes the change trend index of different land use types in the river watershed;$\Delta U_{out}$ represents the sum of the areas converted from a certain land use type to other land use types during the research period; $\Delta U_{in}$ is the sum of the areas for other land use types that were converted into this type during the research period; when ${ - }1 < P \le 0$ the scale of the land use type has decreased and is in a “weak” state and when $0 < P \le 1$ the scale of the land use type has expanded and is in a “rising” state.

### Calculation of the index of landscape type change intensity (*LA*)

The synthetic landscape dynamic attitude index is used to characterize the breadth and depth of the landscape^79^. According to the actual classification of land use types, they were divided into specific sets at four levels: I for unused land; II for water, bush, forestland, grassland; III for paddy field, dry land; and IV for built-up land. This division is represented by the following equation:

3 $$LA = 100 \times \sum\limits_{i = 1}^{n} {A_{i} \times C_{i} }$$

where $LA$ denotes the synthetic land use dynamic degree and varies from 100 to 400,$A_{i}$ is the grade index of grade $i$, $C_{i}$ represents the grade $i$ land area percentage for the entire region, and $n$ is the number of grades, e.g.,$i = 1,2,3,4$.

### Selection and calculation of four landscape metrics

Landscape index is a quantitative research metric used to characterize landscape pattern features and process changes, and to establish associations between patterns and landscape processes. The landscape-level index reflects the overall structural characteristics of the landscape. Here, according to the implications and usefulness of various landscape indices^80,81^, four landscape indices, namely, patch density (*PD*), Shannon’s diversity index *(SHDI*), aggregation index (*AI*), and landscape shape index (*LSI*) were selected and measured by using FRAGSTATS 4.2 software^82^ to characterize the general landscape, and the information for the selected landscape is shown in Table 2. Where, *PD* reflects the complexity of landscape spatial structure and, to some extent, the level of landscape disturbance by human activities; *SHDI* reveals landscape heterogeneity characteristics, and *SHDI* values increase with the increase of landscape patch types and the equalization of its area weight; *AI* describes the aggregation degree among landscapes and reflects the dispersion degree among the same landscape type; *LSI* represents the index of patch shape complexity, the larger the *LSI* index, the more complex the patch shape.

**Table 2:** Selected landscape metrics and their ecological significance.

Item	Abbr	Ecological significance	Mathematical expression	Data range
Patch density	*PD*	Landscape fragmentation	$PD = N/A$	*PD* > 0
Shannon’s diversity index	*SHDI*	Balanced and heterogeneous distribution of different patch types within the region	$SHDI = - \sum\limits_{i = 1}^{n} {P_{i} \ln P_{i} }$	*SHDI* ≥ 0, without limit
Aggregation index	*AI*	Dispersion degree among the same landscape type	$AI = \left[ {\sum\nolimits_{{i = 1}}^{m} {\left( {\frac{{g_{{i_{j} }} }}{{\max \to g_{{ij}} }}} \right)} } \right](100)$	0 ≤ *AI* ≤ 100
Landscape shape index	*LSI*	Plaque complexity	$LSI = \frac{0.25E}{{\sqrt A }}$	*LSI* ≥ 0, without limit

*A* is the total landscape area; *P*_*i*_ is the area proportion of landscape *i; g*_*ij*_ is the number of similar adjacent patches of plaque-type; *E* is the total length of all patch boundaries in the landscape.

### Establishment of a landscape ecological security index system

The landscape ecological security index reflects the impact of natural and human pressures on ecological security from the landscape perspective. In this study, however, the landscape disturbance index (*LDI*) and landscape vulnerability index (*LVI*) was calculated as causal indices to measure landscape ecology based on representative landscape indices^83,84^. In general, the greater the level of disturbance and vulnerability, the lower the level of ecological safety. The landscape disturbance index (*LDI*) is generally reflected by the combined state of patch density (*PD*), fractal dimension (*FRAC*), and Shannon’s diversity index (*SHDI*). The *LDIi* is calculated by the following formula.

4 $$LDIi = \alpha PD + \beta SHDI + \gamma FRAC$$

where the index weights are α, β, and γ, respectively, and they are given values of 0.5, 0.3, and 0.2.

In general, landscape ecological security is related to landscape vulnerability, and the landscape vulnerability index (*LVI*) mainly reflects the degree of variation of each landscape type in the watershed after being disturbed. In turn, there are differences in the degree of disturbance resistance and sensitivity of different landscape types. We assigned vulnerability values to different landscape types: built-up areas 1, forestland and shrubland 2, grassland 3, cultivated land (dryland and paddy field) 4, and water area 4.

Thus, it is possible to construct a landscape ecological security index (*LESI*) based on normalized landscape metrics, which are area-weighted and summed by *LDI* and *LVI* combined. *LESI* is represented by the following equation.

5 $$LESIk = \sum\limits_{i = 1}^{n} {\frac{Aki}{{Ak}}} \times (1 - 10 \times LDIi \times LVIi)$$

where $LESI_{k}$ is the landscape ecological security index of the evaluation unit $k$, $n$ is the number of evaluation units, $A_{ki}$ is the area of landscape type $i$ in the evaluation unit $k$, and $A_{k}$ is the total area of the evaluation unit $k$.
